# A novel signature to predict thyroid cancer prognosis and immune landscape using immune-related LncRNA pairs

**DOI:** 10.1186/s12920-022-01332-7

**Published:** 2022-08-22

**Authors:** Bo Song, Lijun Tian, Fan Zhang, Zheyu Lin, Boshen Gong, Tingting Liu, Weiping Teng

**Affiliations:** Department of Endocrinology and Metabolism, Institute of Endocrinology, National Health Commission Key Laboratory of Diagnosis and Treatment of Thyroid Diseases, The First Hospital of China Medical University, Shenyang, 110001 Liaoning Province People’s Republic of China

**Keywords:** TCGA, lncRNA pairs, Immune infiltration, Thyroid carcinoma, Immune checkpoint inhibitors

## Abstract

**Background:**

Thyroid cancer (TC) is the most common endocrine malignancy worldwide. The incidence of TC is high and increasing worldwide due to continuous improvements in diagnostic technology. Therefore, identifying accurate prognostic predictions to stratify TC patients is important.

**Methods:**

Raw data were downloaded from the TCGA database, and pairwise comparisons were applied to identify differentially expressed immune-related lncRNA (DEirlncRNA) pairs. Then, we used univariate Cox regression analysis and a modified Lasso algorithm on these pairs to construct a risk assessment model for TC. We further used qRT‒PCR analysis to validate the expression levels of irlncRNAs in the model. Next, TC patients were assigned to high- and low-risk groups based on the optimal cutoff score of the model for the 1-year ROC curve. We evaluated the signature in terms of prognostic independence, predictive value, immune cell infiltration, immune status, ICI-related molecules, and small-molecule inhibitor efficacy.

**Results:**

We identified 14 DEirlncRNA pairs as the novel predictive signature. In addition, the qRT‒PCR results were consistent with the bioinformatics results obtained from the TCGA dataset. The high-risk group had a significantly poorer prognosis than the low-risk group. Cox regression analysis revealed that this immune-related signature could predict prognosis independently and reliably for TC. With the CIBERSORT algorithm, we found an association between the signature and immune cell infiltration. Additionally, immune status was significantly higher in low-risk groups. Several immune checkpoint inhibitor (ICI)-related molecules, such as PD-1 and PD-L1, showed a negative correlation with the high-risk group. We further discovered that our new signature was correlated with the clinical response to small-molecule inhibitors, such as sunitinib.

**Conclusions:**

We have constructed a prognostic immune-related lncRNA signature that can predict TC patient survival without considering the technical bias of different platforms, and this signature also sheds light on TC’s overall prognosis and novel clinical treatments, such as ICB therapy and small molecular inhibitors.

**Supplementary Information:**

The online version contains supplementary material available at 10.1186/s12920-022-01332-7.

## Introduction

In recent decades, the incidence of thyroid cancer (TC) has been increasing steadily over the past 30 years worldwide [1]. With the development of diagnostic technology, the detection rate of TC in the world has increased year by year, ranking fifth in the incidence of malignant tumors [2], and the growing incidence of TC is raising serious public health issues worldwide. Even though most TCs have a relatively good prognosis, approximately 10% of patients with differentiated thyroid cancer (DTC) may progress to invasive disease, 5% progress to distant metastasis, and approximately 20–30% may relapse [3].

The immune system is now considered to have an important role in the elimination of cancer cells and sheds light on the mechanisms of cancer–immune evasion, contributing to tumor outgrowth [4, 5]. Although immunosurveillance prevents the development of most tumors in normal individuals, cancer cells can still deploy escape strategies such as initiating immune checkpoints [6]. Immune checkpoint inhibitors (ICIs) can restore the normal work of the immune system by suppressing the signal of "rest from work", which is sent by immune checkpoints, and then launch an attack on tumor cells [7]. Immune checkpoint blockade (ICB) immunotherapy has shown durable responses and improved clinical outcomes for patients across most malignancies, including thyroid cancer. Growing evidence has proven that ICI-related genes such as PD-1 and PD-L1 are expressed in ATC and DTC [8]. Pembrolizumab, an anti-PD-1 antibody, may be effective for patients with TC because of the KEYNOTE-158 trial, which shows that approximately 60% of patients in this program achieved disease control [9].

In addition, several studies have demonstrated that tyrosine kinase inhibitors (TKIs) can prevent the proliferation and tumorigenicity of thyroid cancer cells. Treatment with small-molecule tyrosine kinase inhibitors, including gefitinib, pazopanib, lenvatinib, and axitinib, are approved by the FDA to treat thyroid cancer [10]. Some clinical trials were performed to evaluate the efficacy and safety of these inhibitors [11]. Nevertheless, TKIs are promising new agents for the treatment of patients with thyroid cancer.

Long noncoding ribonucleic acids (lncRNAs) belong to the family of noncoding RNAs and are functionally defined as transcripts > 200 nucleotides in length with no protein-coding potential [12]. In the past few years, lncRNAs have attracted much attention due to their previously underappreciated transcriptional regulation, and they can function as both oncogenes and tumor suppressors [13]. It is clear that lncRNAs play crucial roles in the regulation of various biological and pathological behaviors of malignant tumors, especially in tumorigenesis and progression [14, 15].

Moreover, a growing body of studies has shown that the expression of lncRNAs is linked to the immune response and tumor progression. In addition, lncRNAs have an essential role in the development of immune cells and in pathogen response pathways. Notably, individual lncRNAs can act functionally through modular domains and often link protein activity to DNA or RNA targets through interactions with both [16]. Aberrant lncRNA expression has been observed in various cancers [17]. Recently, an increasing number of lncRNAs have been discovered to play an important role in TC tumorigenesis and development. Peng et al. [18] summarized representative lncRNAs in thyroid cancer, such as NEAT1, HOTAIR, PTCSC2, GAS8-AS1, MEG3, BANCR, GAS5, and MALAT1 et al., which are highly linked to the biological behavior of thyroid cancer and show significant value in diagnosis and treatment. Recent study showed that the expression level of lncRNA n384546 was upregulated in TC patients and that its interference inhibited cancer cell proliferation, invasion, and migration [19]. Reliable prognostic models related to tumor immune infiltration may affect the diagnosis, evaluation and treatment decisions of tumors. LncRNAs have recently been used to establish prognostic signatures. Huang et al. [20] constructed an immune-related lncRNA signature to predict the survival outcome of patients with breast cancer. Xu et al. [21] built a signature based on seven immune-related lncRNAs, which showed reliable prognostic value in hepatocellular carcinoma and may predict the outcome of immune checkpoint blockade (ICB) therapy.

In this study, we established a novel method to construct a predictive signature for TC based on immune-related lncRNA pairs, which did not need to consider the technical bias of different platforms. Next, to explore the latent role of this signature, we integrated the immune model with clinical factors of TC patients to build a composite prognostic index, which allowed improved estimation of prognosis. We also explored the correlation between the signature and several aspects, such as immune cell infiltration, immunosuppressive biomarkers, and small-molecule tyrosine kinase inhibitor efficacy. Overall, our new model might provide insight into the prognosis and clinical treatment of TC.

## Materials and methods

### Data download and preprocessing and differential expression analysis

The gene expression data and clinical data of TC patients were downloaded from The Cancer Genome Atlas (TCGA, http://cancergenome.nih.gov/) to perform a comprehensive analysis. The RNA-seq profiles of 558 cases comprised 500 thyroid carcinoma samples and 58 normal thyroid samples. We also included clinical data from 504 TC patients with an overall survival time of 0 days. Samples from the TCGA database were divided randomly into a training set (n = 252) and an internal test set (n = 252) at a ratio of 1:1. Next, the data were annotated to distinguish the mRNAs and lncRNAs for further analysis. We retrieved 2483 immune-related genes (IRGs) from the ImmPort database (https://immport.niaid.nih.gov) and performed Pearson correlation to identify the association between immune-modulating genes and all lncRNAs. LncRNAs with a correlation coefficient |R|≥ 0.4 and *P* ≤ 0.001 were considered related to immune genes and were used for further analysis. Screening of the DEirlncRNAs was based on logFC > 1 and FDR < 0.05 using the limma R package [22].

### Pairing immune-related lncRNAs

We performed cyclical pairwise comparisons between the DEirlncRNA expression values to generate a score for each irlncRNA pair and established a 0-or-1 matrix. If the expression level of lncRNA1 was higher than that of lncRNA2, this pair was assigned a score of 1; otherwise, the score was 0. To prevent biases and unrepeatability, we validated the expression quantities of the lncRNA pairs. After removing lncRNA pairs scoring 0 or 1 in more than 80% and less than 80% of the total pairs, the remaining pairs were considered valid matches to build the prognostic signature.

### Construction of a novel signature based on the DEirlncRNA pairs

Univariate Cox regression analysis (*P* < 0.001) was first implemented to identify the prognostic irlncRNA pairs with TC by using R (survival package). Subsequently, least absolute shrink age and selection operator (LASSO) Cox regression was conducted by utilizing the Glmnet R package to reduce the number of pairs [23]. Lasso regression was carried out to acquire a well-balanced prognostic model by running 1000 cycles. Then, 14 pairs of immune-related lncRNAs were ultimately identified as our prognostic model, and the risk score of each TC patient was calculated based on the following formula: RiskScore = . Here, n was the number of selected lncRNA pairs, β_i_ was the coefficient of pair i, and P_i_ was the expression of lncRNA pair i. To validate the sensitivity and accuracy of the model, time-dependent ROC curve analysis was performed by utilizing the SurvivalROC package in R. The AUC was calculated for ROC curves, and sensitivity and specificity were calculated to assess the performance of the risk score. The optimal cutoff score based on the 1-year ROC curve was identified to separate the patients into low-risk and high-risk groups.

### Reverse-transcription quantitative PCR

To validate the bioinformatics analysis results, 14 single lncRNAs of 14 DEirlncRNA pairs were selected to perform quantitative real-time PCR (qRT‒PCR) analyses. Twelve matched tumor and peritumor samples of PTC were collected from the First Affiliated Hospital of China Medical University. Total RNA was extracted from tissue samples using TRIzol (Invitrogen, United States), and then RNA was reverse transcribed into cDNA with the QuantiTect Reverse Transcription Kit (Takara, Shiga, Japan). Gene expression was analyzed by qRT‒PCR, which was performed with SYBR Premix Ex TaqII (Takara) and a LightCycler 480 system (Roche, Indianapolis, IN, USA). GAPDH was used for data normalization, and these data were analyzed by 2−ΔΔCT. The primer sequences are listed in Additional file 1: Table S1.

### Validation of the risk assessment model

We estimated the prognostic capability of the risk score, sex, age and tumor stage (TNM stage and clinical stage) for overall survival in terms of time-dependent AUC values. The survival difference between subgroups was evaluated by Kaplan‒Meier survival analysis. The survival curve was visualized using the survival and survminer packages in R. The specific risk score value of each sample based on the signature was also used for visualization in R. We further performed principal component analysis (PCA) to assess the accuracy of the classification according to different risk scores. To predict the reliability and stability of the constructed model, Pearson’s χ2 test was used to assess the association between the signature and clinical features. The risk score differences between groups of conventional clinical features were compared by the Wilcoxon signed rank test. The analysis results were visualized by a strip chart and box diagrams. Moreover, we used univariate and multivariate Cox proportional hazards analyses of the risk score and other clinical characteristics to examine whether the model could be used as an independent variable. Then, the prognostic model was further validated in the internal validation set, including the training set and test set.

### Correlation between the risk model and immune cell infiltration

The CIBERSORT algorithm was performed to estimate the tumor-infiltrating immune cell profiles of the samples in the TC dataset, followed by quality filtering, and only 509 tumor samples with *P* < 0.05 were selected for the following analysis. To further explore whether the risk model was related to immune cell infiltration in TC, other methods, such as TIMER, XCELL, QUANTISEQ, MCP-counter, EPIC, and CIBERSORT-ABS, were also used. We applied the Wilcoxon signed-rank test to compare the tumor-infiltrating immune cells among different risk groups. A box chart was used to visualize the results. To assess the relationship between the model and the infiltrated immune cells, Spearman correlation analysis was performed. The results of the Spearman rank correlation coefficients are shown in the lollipop-style mutation diagram. We visualized the results using the ggplot2 package in R.

### Correlation between the risk model and the molecules related to ICIs

An ESTIMATE algorithm [24] was used to estimate the proportion of stromal cells (stromal score) and immune cells (immune score) that infiltrated the tumor tissue between low-risk and high-risk cases. We also compared the expression of the major markers of cytolytic activity (GZMA,PRF1) and the HLA gene between the high- and low-risk groups. The limma package was applied to explore the expression of ICI-related molecules among the different risk groups, and the results are shown in violin plots developed using the ggpubr package in R.

### Analyses of the model ability in clinical treatment

Small-molecule inhibitors such as gefitinib, pazopanib, lenvatinib, axitinib, AMG-706 (also known as motesanib), tipifarnib, sunitinib, and sorafenib, which are now approved by the FDA, have shown great curative effects in thyroid cancer. To evaluate the ability of the signature to treat thyroid cancer, we calculated the IC50 values of these drugs in the THCA dataset. We performed the Wilcoxon signed-rank test to analyze the differences among groups, and the results are displayed in box plots utilizing the pRRophetic, ggpubr, and ggplot2 R packages.

## Results

### Identification of differentially expressed irlncRNAs (DEirlncRNAs)

**Fig. 1:**
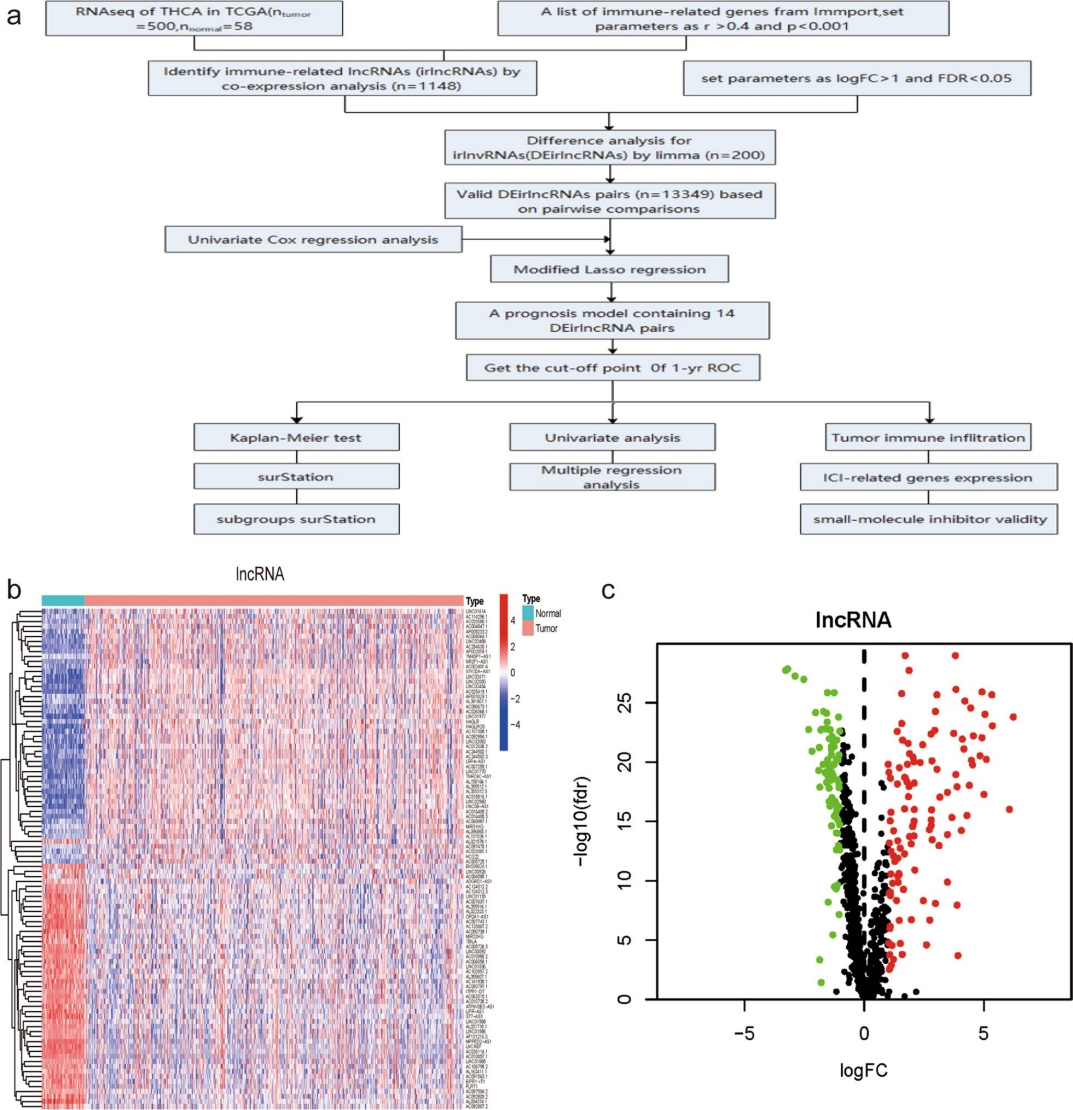
Identification of immune-related lncRNAs in TC. (**a**) Analysis workflow of this study. (**b**) The heatmap shows the expression level of differentially expressed immune-related lncRNAs (DEirlncRNAs) in the TCGA dataset (blue, lower expression; red, higher expression). (**c**) The volcano plot represents the result of DEirlncRNA analysis of the THCA dataset (green: downregulated genes; black: no differentially expressed genes; red: upregulated genes)

This study was carried out based on the flowchart shown in Fig. 1a. First, RNA sequencing (RNA-seq) transcriptome data of TC were downloaded from the TCGA database. A total of 505 available TC patients were included in our study, and the baseline characteristics of all the patients are shown in Table 1. A total of 1148 irlncRNAs were identified by performing a coexpression analysis (shown in Additional file 2: Table S2). Next, we set the thresholds as the log fold change (FC) > 1.0 or < -1.0 and false discovery rate (FDR) < 0.05 and identified 200 DEirlncRNAs between tumor and normal thyroid tissues (Fig. 1b), including 117 upregulated and 83 downregulated genes (Fig. 1c).

**Table 1:** Clinical characteristics of the thyroid cancer patients

Characteristic	Type	n	Proportion (%)
Age	< 55	339	67.26
	> = 55	165	32.74
Gender	Female	368	73.02
	Male	136	26.98
Stage	Stage I	399	79.17
	Stage II	77	15.28
	Stage III	22	4.36
	Stage IV	6	1.19
T stage	T1	144	28.57
	T2	167	33.13
	T3	170	33.73
	T4	23	4.57
M stage	M0	496	98.41
	M1	8	1.59
N stage	N0	272	53.97
	N1	232	46.03

### Construction of a novel prognostic signature based on DEirlncRNA pairs for TC

**Fig. 2:**
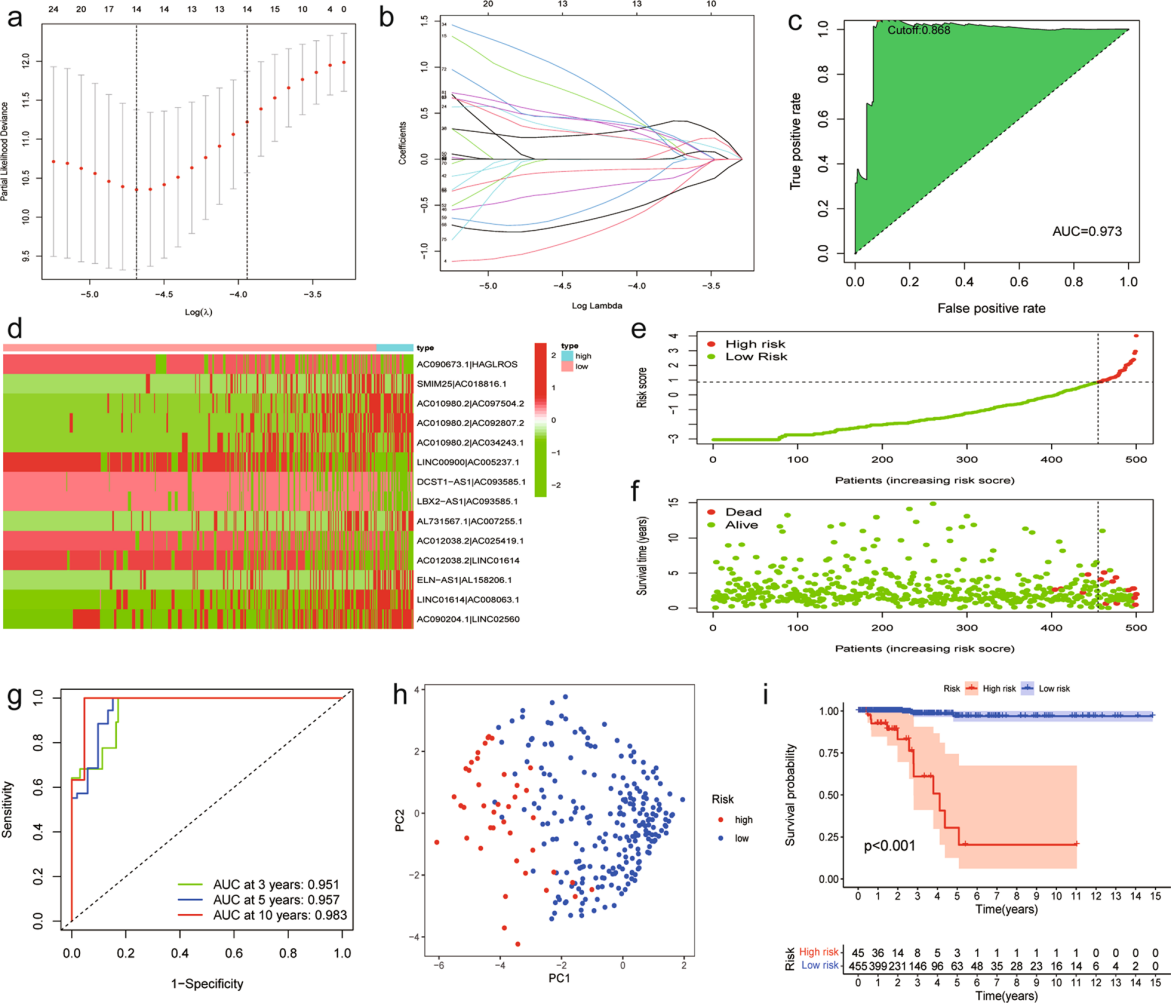
Construction of a risk assessment model and confirmation of the signature. (**a**, **b**) Results of the lasso regression. (**c**) The cutoff value of the one-year ROC curve was used to separate the patients. (**d**) Fourteen DEirlncRNA pairs were identified to construct the signature. (**e**) Distribution of lncRNA model risk scores. (**f**) Survival status of TC patients in the subgroups. (**g**) ROC analysis of the risk scores for overall prognosis prediction. The 3-(red), 5-(green), and 10-year (blue) ROC curves of the model suggested that all AUC values were over 0.95. (**h**) PCA plot of the lncRNA model. (**i**) Kaplan‒Meier curve presenting survival in the high-risk and low-risk sets

First, a total of 200 DEirlncRNAs were screened by pairwise comparison, and 13,349 DE-irlncRNA pairs were constructed. With single factor Cox regression analysis, prognosis-related DE-irlncRNA pairs were further selected. Then, lasso-penalized Cox analysis was conducted to narrow the number of lncRNA pairs to 14 over 1000 repetitions (Fig. 2a, b). Next, we constructed a risk assessment model that contained these 14 DEirlncRNA pairs using a risk score method and calculated the risk score of each sample. We performed receiver operating characteristic (ROC) analysis at 1 year (area under the curve (AUC) = 0.973) and identified the optimal cutoff point at 0.868 to divide 497 patients into high- and low-immune risk groups (Fig. 2c, d). We further investigated the prognostic value of the signature by plotting the distributions of the risk score and survival time (Fig. 2e, f). Low-risk TC patients exhibited a superior clinical outcome compared with high-risk TC patients. Then, 3-, 5-, and 10-year ROC curves were plotted and showed that the model had the ability to predict the survival outcome of TC patients with high accuracy and sensitivity (Fig. 2g).

### Expression levels of fourteen DEirlncRNAs

We further validated the expression of 14 single lncRNAs of DEirlncRNA pairs in 12 matched tumor and peritumor samples using qRT‒PCR analysis. Compared with the peritumor controls, the expression levels of LINC00900, DCST1-AS1, HAGLROS, LINC02560, AL158206.1, SMIM25, AC090673.1, AC012038.2, and LINC01614 was significantly increased (*P* < 0.05), whereas ELN-AS1, AC093585.1, AC005237.1, and LBX2-AS1 were significantly decreased in PTC tissues (*P* < 0.05) (Additional file 3: Fig. S1), which was consistent with the bioinformatics results obtained from the TCGA dataset.

### Clinical assessment and evaluation of the signature

**Fig. 3:**
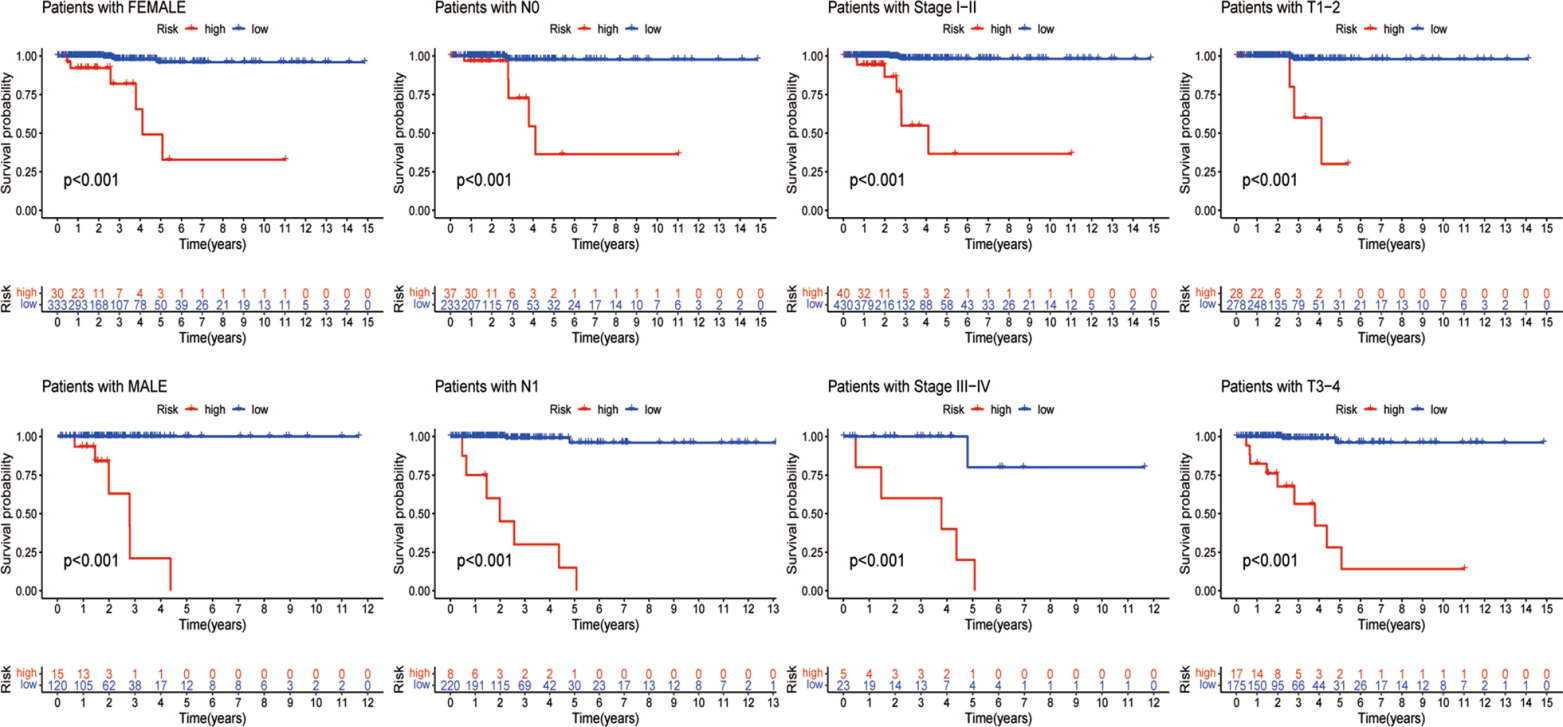
The prognostic value of the signature. THCA patients were assigned to different subgroups, including sex (male and female), N stage (N0 and N1), stage (stage I + II and stage III + IV) and T stage (T1 + T2 and T3 + T4)

Based on the validated optimal cutoff point, 45 TC patients with higher risk scores were included in the high-risk group, and 452 TC patients with lower risk scores were included in the low-risk group. The PCA results confirmed the reliability of this kind of classification (Fig. 2h). Kaplan‒Meier survival analysis showed that low-risk TC patients exhibited a significantly better prognosis than high-risk TC patients (*P* < 0.001, Fig. 2i). Furthermore, we assigned THCA patients into different subgroups according to clinical characteristics, including sex (male and female), N stage (N0 and N1), stage (stage I + II and stage III + IV) and T stage (T1 + T2 and T3 + T4). KM analysis also showed that high-risk patients had a worse overall survival than low-risk patients, which indicated that the signature prediction was excellent (*P* < 0.001, Fig. 3).

### Signature as an independent prognostic predictor

**Fig. 4:**
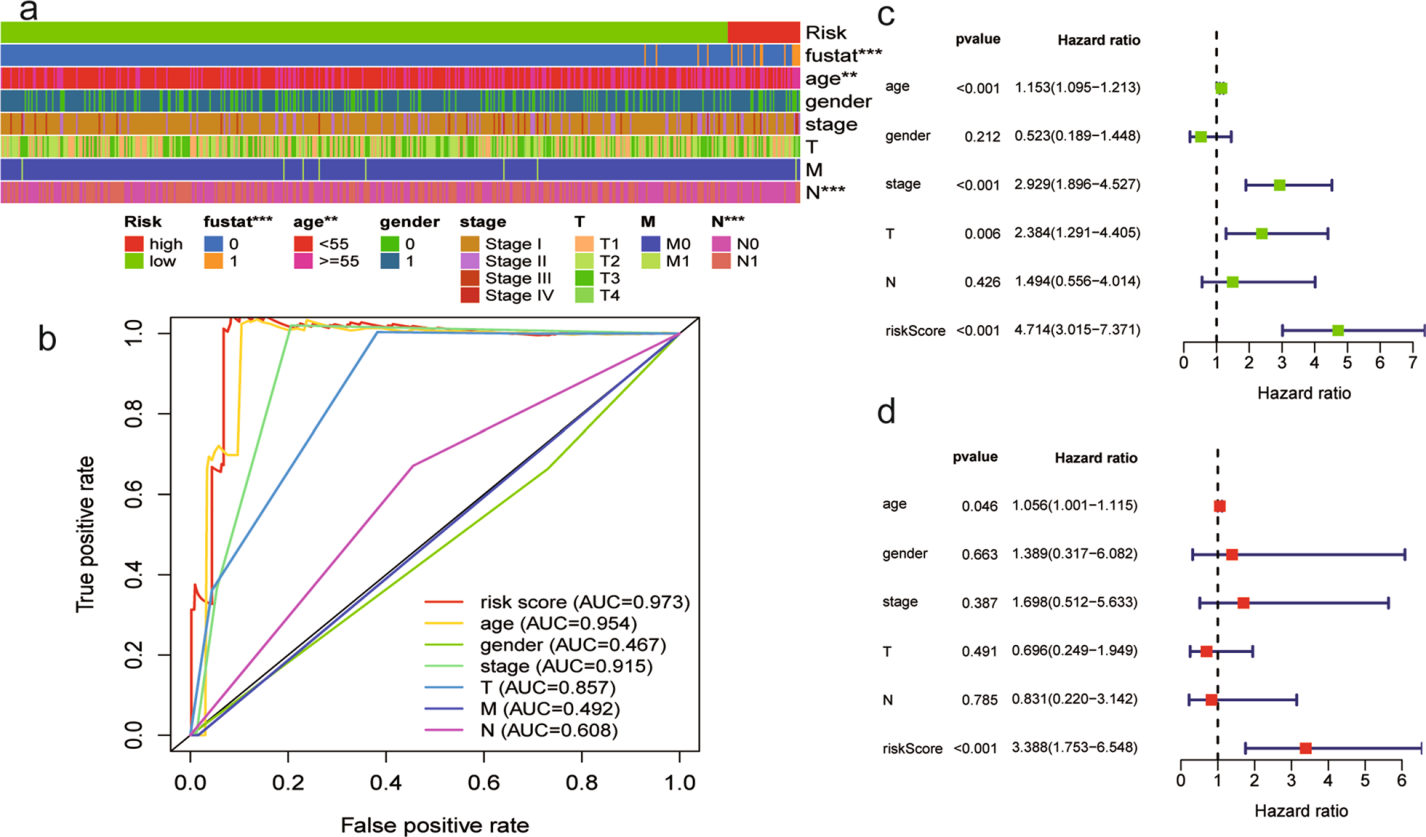
The clinical value of the signature. (**a**) Strip chart, which was labeled as follows: < 0.001 = ***, < 0.01 = **, and < 0.05 = *. (**b**) Comparison of the 1-year ROC curves of the risk score with those of other clinical features showed the superiority of the risk score. (**c**, **d**) Univariate and multivariate Cox regression analyses were applied to identify prognostic factors associated with TC

A series of chi-square tests were performed to explore the relationship between the risk of TC and common clinical features, including age, sex, TNM stage, and clinical stage. The strip chart (Fig. 4a) and scatter diagrams showed that age, N stage, clinical stage, and survival status (Additional file 4: Fig. S2) were significantly related to the risk of TC. Furthermore, we compared the AUCs of the 1-year ROC curves of the risk score to those of other clinical features and found that the risk score achieved a higher AUC value among these factors (Fig. 4b). Next, univariate and multivariate Cox regression analyses were performed to validate prognostic factors associated with TC. Univariate analysis showed that age (*P* < 0.001, hazard ratio (HR) = 1.153, 95% confidence interval (CI) [1.095 ~ 1.213]), stage (*P* < 0.001, HR = 2.929, 95% CI [1.896–4.527]), T stage (*P* = 0.005, HR = 2.384, 95% CI [1.291–4.405]), and risk score (*P* < 0.001, HR = 4.714, 95% CI [3.015–7.371]) were significantly associated with prognosis, whereas only age (*P* = 0.040, HR = 1.061, 95% CI [1.003–1.122]) and risk score (*P* = 0.005, HR = 3.409, 95% CI [1.750 − 6.641]) independently predicted the clinical outcome of TC patients by multivariate Cox regression analysis (Fig. 4c, d, Table 2).

**Table 2:** Independence of the signature for predicting the clinical outcomes of TC

Id	Univariate analysis			Multivariate analysis		
	HR	95%CI	P	HR	95%CI	P
Age	1.153	1.095 ~ 1.213	< 0.001	1.061	1.003 ~ 1.122	0.040
Gender	0.523	0.189 ~ 1.448	0.212	1.601	0.330 ~ 7.768	0.559
Stage	2.929	1.896 ~ 4.527	< 0.001	1.286	0.290 ~ 5.709	0.741
T	2.384	1.291 ~ 4.405	0.005	0.779	0.264 ~ 2.304	0.652
M	2.731	0.359 ~ 20.794	0.332	2.701	0.119 ~ 61.347	0.652
N	1.494	0.556 ~ 4.014	0.426	0.533	0.235 ~ 3.618	0.908
Riskscore	4.714	3.015 ~ 7.371	< 0.001	3.409	1.750 ~ 6.641	< 0.001

(a) The univariate analysis illustrated the clinicopathological factors related to TC prognosis. (b) The multivariate analysis revealed the clinicopathological factors related to TC prognosis

### Internal validation of the signature

We further validated the prognostic model in the training (Additional file 5: Fig. S3) and testing sets (Additional file 6: Fig. S4). The results from two Kaplan‒Meier survival analyses indicated that the risk score was closely linked to OS (*P* < 0.001) in the training and testing sets. Moreover, the PCA revealed a significant disparity in patients between the high- and low-risk groups. We next performed a time-dependent ROC analysis in the training cohort, and the results showed an area under the curve (AUC) of 0.929, 0.956, and 0.951 at 1 year, 3 years, and 5 years, respectively. In the testing cohort, the predictive efficacy (AUC values of 0.976, 0.942, and 0.981, respectively) was validated similarly. Univariate and multivariate Cox regression analyses showed that the risk score could independently predict the clinical outcome of TC patients in both sets. This result confirmed the overall accuracy and validity of the prognostic signature.

### Correlation of the risk assessment model with immune cell infiltration in TC

**Fig. 5:**
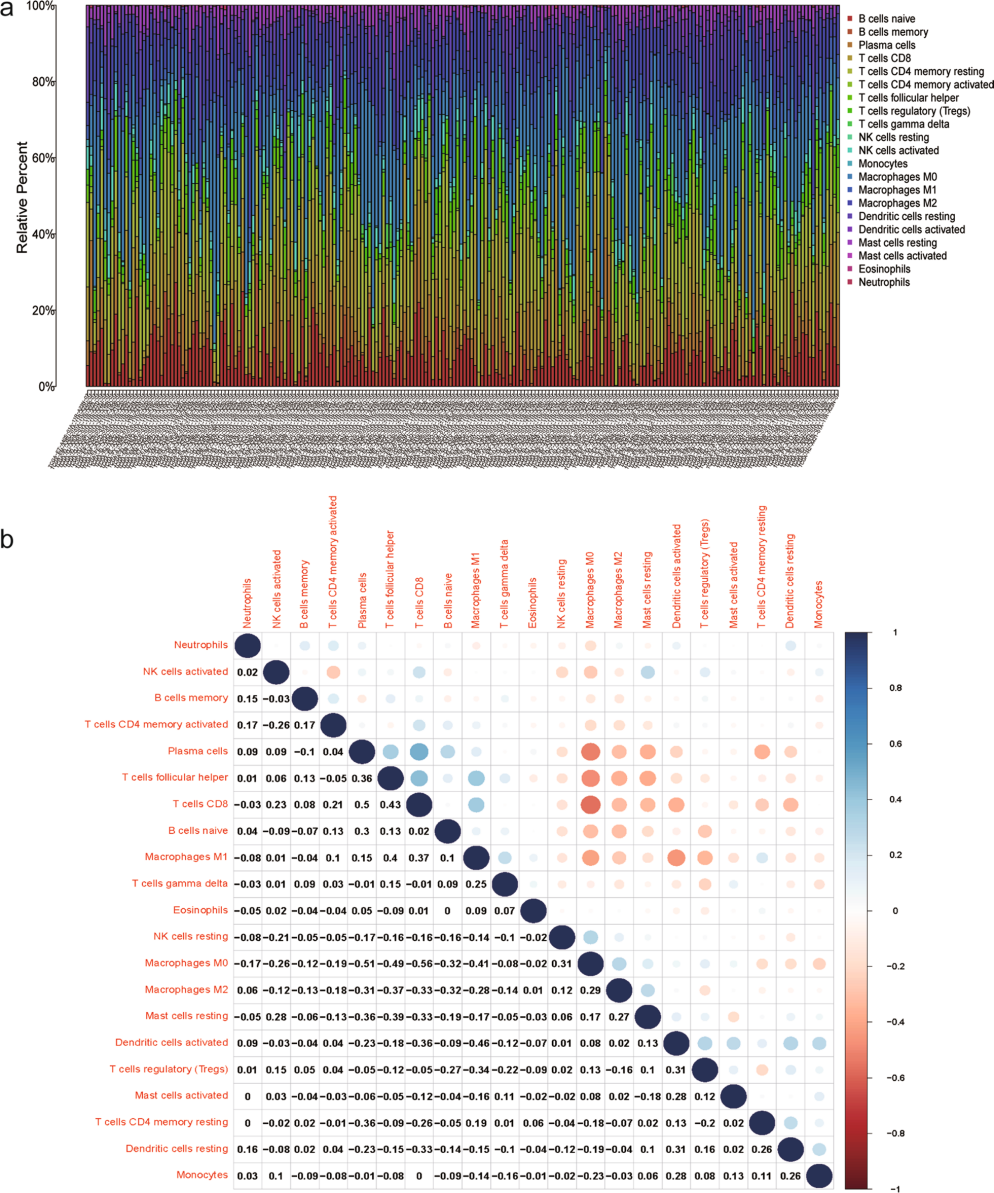
TIC profile in tumor samples and correlation analysis. (**a**) Proportions of the 21 kinds of TICs explored by the CIBERSORT algorithm. (**b**) Correlations between 21 immune cell components

**Fig. 6:**
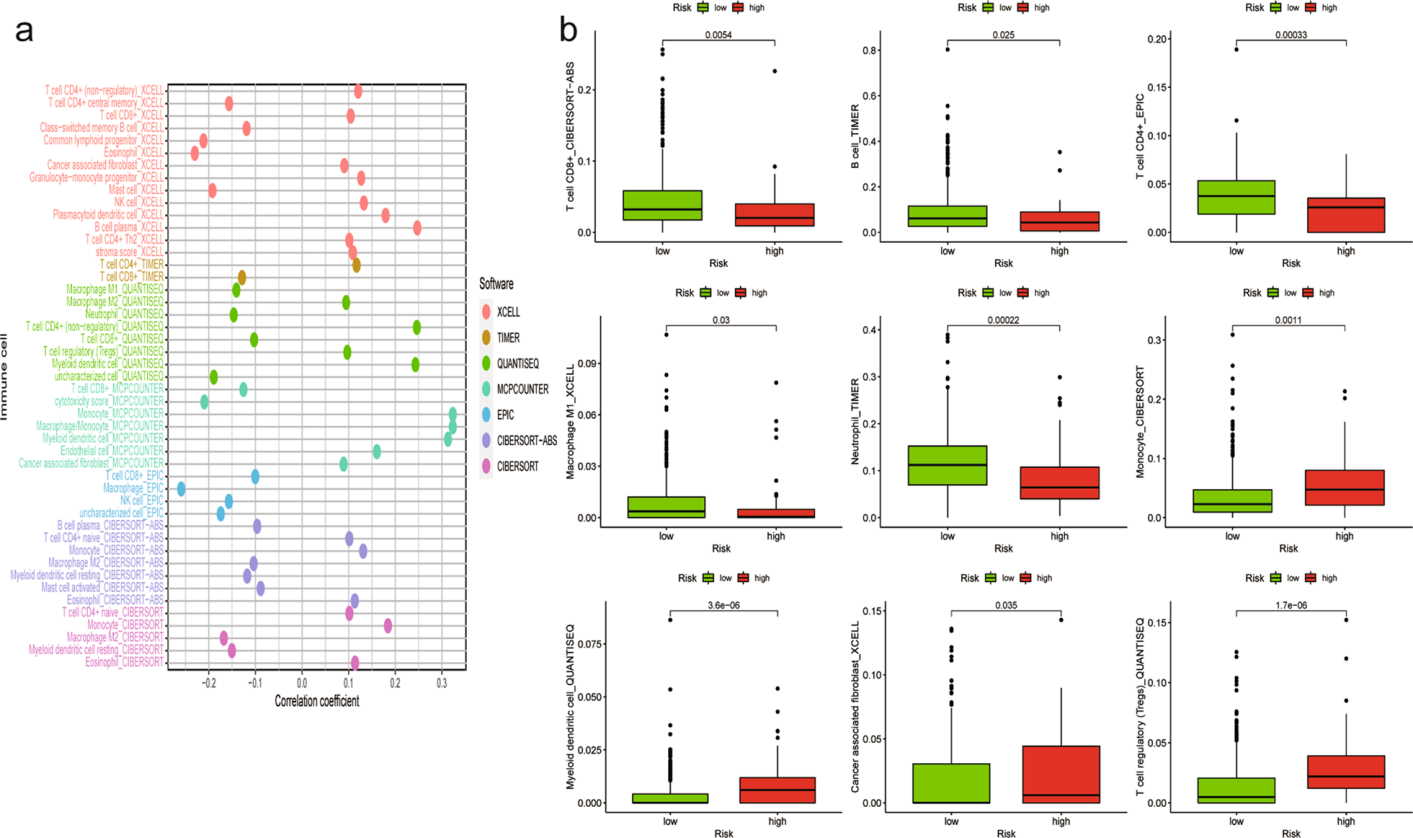
Immune infiltration status in different risk groups. **a** Correlation between tumor immune infiltration and the immune-related lncRNA signature. **b** The *P* value comparing the risk score and tumor-infiltrating immune cells

The TME is essential for cancer prognosis and is composed of tumor cells, stromal cells, and infiltrating immune cells. We next investigated whether the signature was associated with the TME in TC patients. The proportion of tumor-infiltrating immune subsets was determined based on the CIBERSORT algorithm, and 21 kinds of immune cell profiles were detected in the TC samples (Fig. 5a, b). We found that the risk score had a significant relationship with the infiltration levels of immune cells. Correlation analysis was conducted using the Spearman correlation test, and the results were displayed in a lollipop diagram (Fig. 6a). The data are listed in Additional file 7: Fig. S5. The high-risk samples showed a negative association with tumor-infiltrating immune cells, such as CD8 + T cells, CD4 + T cells, neutrophils, B cells, NK cells and M1 macrophages, compared with the low-risk samples, whereas they were positively associated with Tregs, myeloid dendritic cells, monocytes, and cancer-associated fibroblasts, as analyzed by the Wilcoxon signed-rank test (Fig. 6b, Additional file 8: Table S3).

### Assessment of immune status in the THCA subtypes

**Fig. 7:**
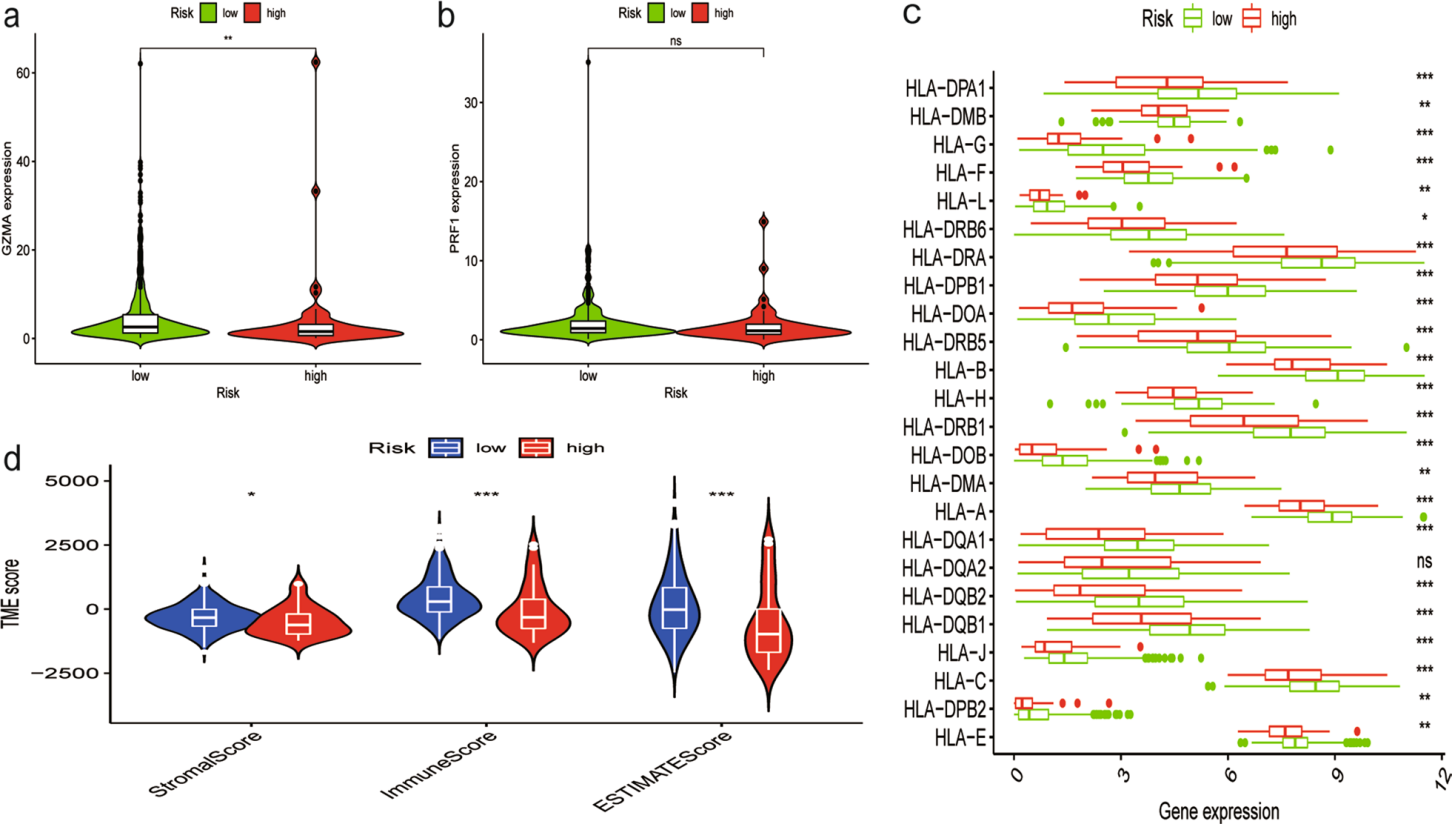
Differential immune features in the high-risk and low-risk subgroups. (**a**, **b**) The gene expression of GZMA and PRF1 in high- and low-risk groups. (**c**) The expression of HLA-related genes in high- and low-risk groups. (**d**) Stromal Score, Immune Score and ESTIMATE Score in the two risk subgroups (< 0.001 = ***, < 0.01 = **, and < 0.05 = *)

We further evaluated the transcript levels of granzyme A (GZMA) and perforin (PRF1) in different risk groups. We found increased expression of GZMA in the low-risk group compared with the high-risk group (*P* < 0.01, Fig. 7a), whereas PRF1 (*P* > 0.05, Fig. 7b) showed no significant difference. The levels of human leucocyte antigen (HLA)-related gene expression were further studied, as shown in Fig. 7c. The results suggested that the low-risk group had higher expression levels of HLA genes than the high-risk group. Through the ESTIMATE algorithm, we explored the different characteristics of the immune microenvironment in the two risk groups. Compared with the high-risk group, the ESTIMATE score, immune score, and stromal score were all higher in the low-risk group (Fig. 7d). The results indicated that this phenomenon might be associated with the immunosuppressive microenvironment.

### Correlation of the signature with genes related to ICIs in TC

**Fig. 8:**
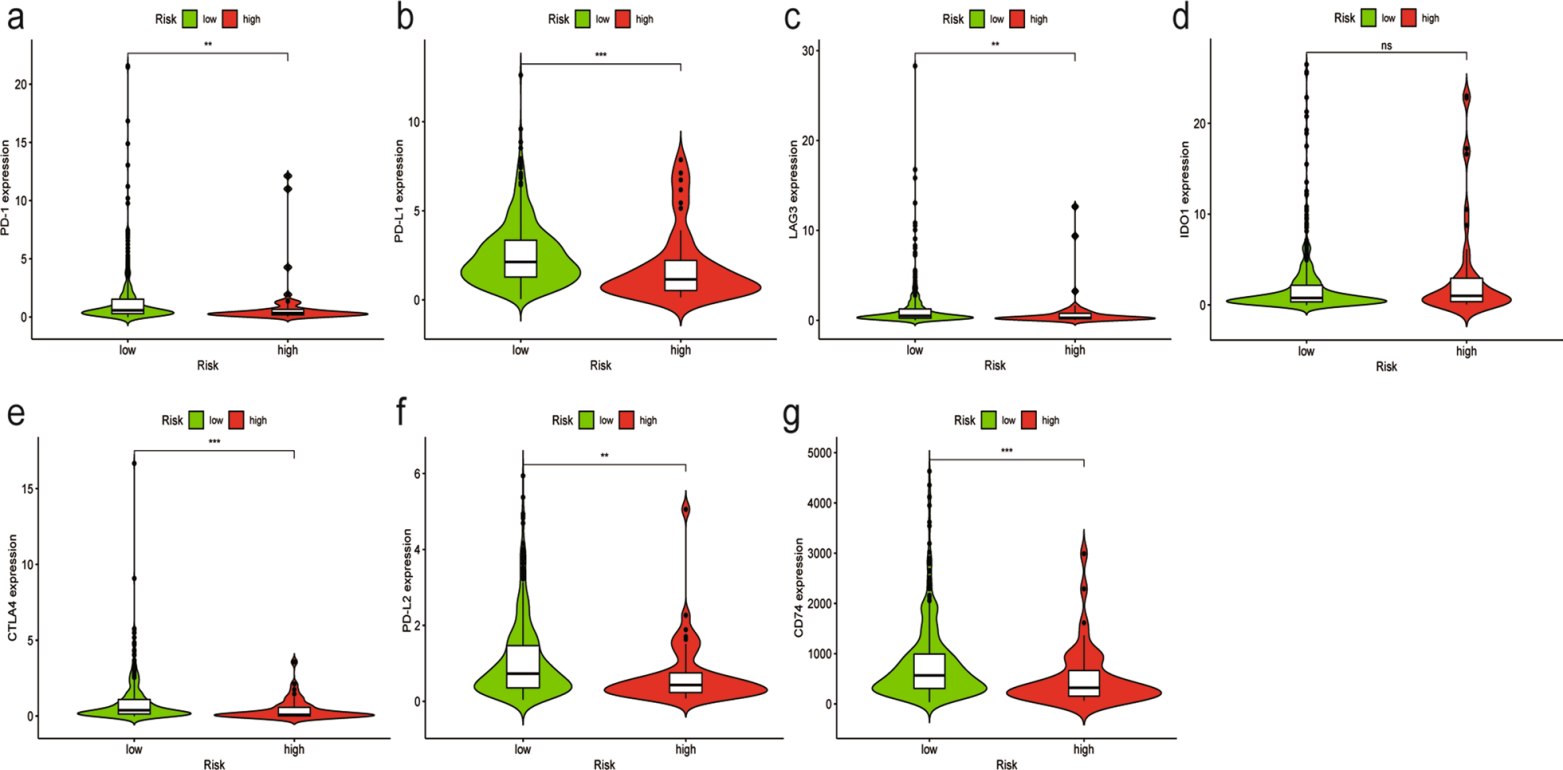
Correlation between ICI target genes and the immune-related lncRNA signature. (**a**–**g**) The high-risk score group showed a negative correlation with the expression of PD-1 (**a**), PD-L1 (**b**), LAG3 (**c**), CTLA-4 (**e**), PD-L2 (**f**), and CD74 (**g**), whereas IDO1 (**d**) showed no significant difference. (< 0.001 = ***, < 0.01 = **, and < 0.05 = *)

Immune checkpoint blockade has revolutionized cancer treatment, and ICB therapy has already been applied in thyroid cancer patients. Therefore, we employed seven key ICI-related genes, PDCD1 (also called PD-1), CD274 (also called PD-L1), PDCD1LG2 (also called PD-L2), CTLA-4, LAG3, CD74, and IDO1, to investigate whether these ICI-related molecules were related to our new signature. The results indicated that the high-risk score group showed a negative correlation with the expression of PD-1 (*P* < 0.01, Fig. 8a), PD-L1 (*P* < 0.001, Fig. 8b), LAG3 (*P* < 0.01, Fig. 8c), CTLA-4 (*P* < 0.001, Fig. 8e), PD-L2 (*P* < 0.05, Fig. 8f), and CD74 (*P* < 0.001, Fig. 8g), whereas IDO1 (*P* > 0.05, Fig. 8d) showed no significant difference.

### The signature as an indicator in TC treatment

**Fig. 9:**
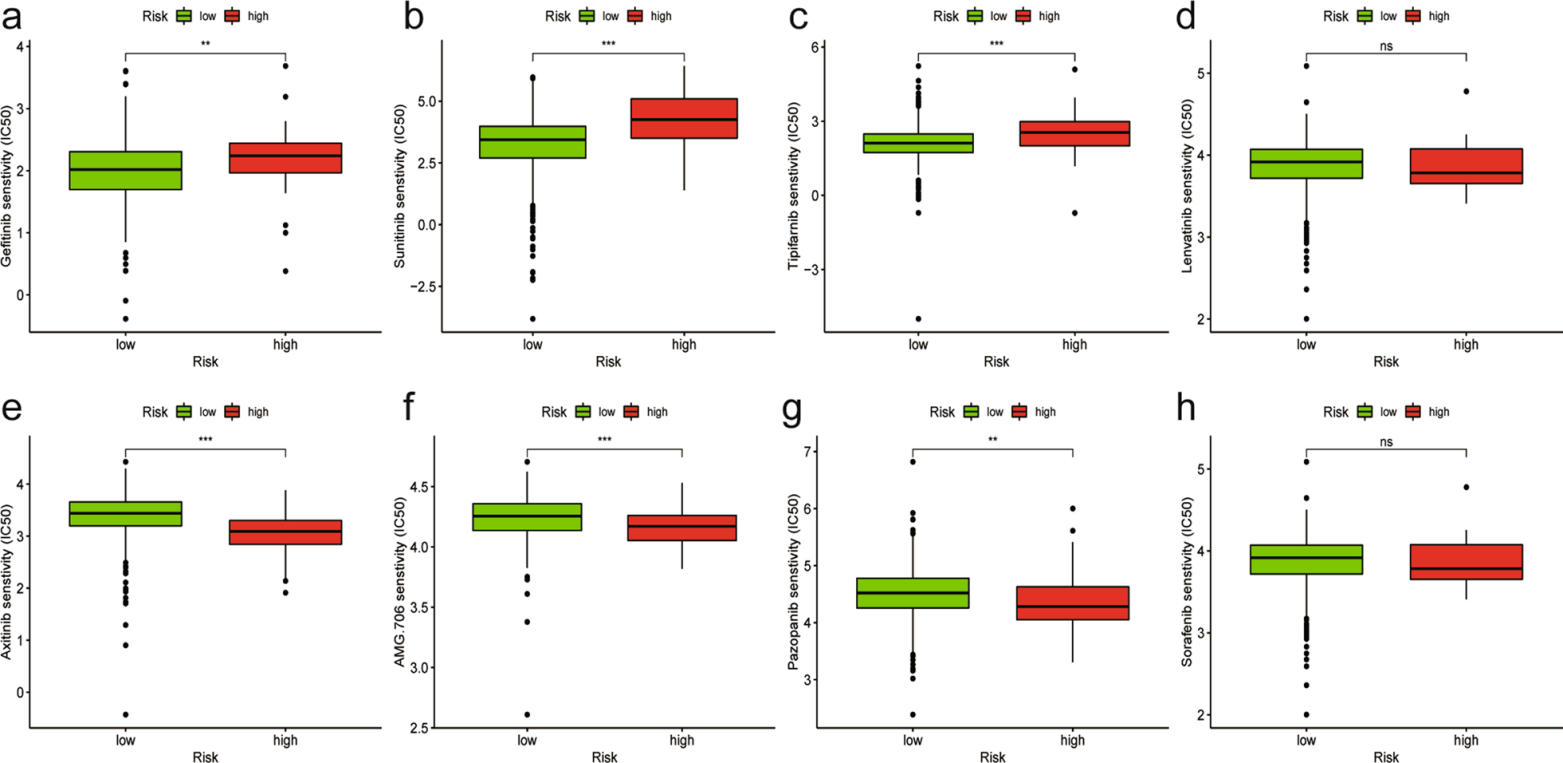
Correlation between common TKIs and the immune-related lncRNA signature. (**a**–**h**) The high-risk samples were positively related to the IC50 of gefitinib (**a**), sunitinib (**b**), and tipifarnib (**c**) but negatively related to axitinib (**e**), AMG-706 (**f**), and pazopanib (**g**). Lenvatinib (**d**) and sorafenib (**h**) had no relationship with the signature. (< 0.001 = ***, < 0.01 = **, and < 0.05 = *)

As a personalized medical treatment, small-molecule inhibitors have been widely applied in the clinical treatment of thyroid cancer. We next investigated the relationship between the signature and the half maximal inhibitory concentration (IC50) of some common inhibitors in the THCA dataset. As shown in Fig. 9, the high-risk samples were positively related to the IC50 of gefitinib (*P* < 0.01, Fig. 9a), sunitinib (*P* < 0.001, Fig. 9b), and tipifarnib (*P* < 0.001, Fig. 9c) but negatively related to axitinib (*P* < 0.001, Fig. 9e), AMG-706 (*P* < 0.001, Fig. 9f), and pazopanib (*P* < 0.01, Fig. 9g). In addition, lenvatinib and sorafenib showed no relationship with the signature (*P* > 0.05, Fig. 9d, h).

## Discussion

Thyroid cancer is the most common type of cancer of the endocrine system, and its incidence has increased almost threefold over the past decades [25]. Traditional clinical characteristics, such as TNM stage, can be used to predict the severity related to TC, but it is difficult to accurately estimate the risk of recurrence [26]. Thus, it is imperative to establish powerful tools that can be effectively applied to aid in the diagnosis, prognosis, and treatment of patients with TC.

Accumulating evidence reveals that since lncRNAs have high tissue and cancer specificity, they might play an active role in cancer initiation, development and progression. An increasing number of studies have shown that lncRNAs promote tumor cell proliferation, invasion, metastasis, and angiogenesis and can serve as an excellent tool to modulate therapeutic decisions in cancer. Increasing evidence suggests that lncRNAs are involved in TC tumorigenesis and progression as important regulatory factors [27]; thus, lncRNAs have attracted much attention as potential targets in the diagnosis and treatment evaluation of TC. For example, Liu et al. found that MALAT1 may have an oncogenic function in PTC and may thus be a potential diagnostic marker for PTC [28]. In our research, a few of the DEirlncRNAs in the model have already been revealed to play roles in various cancers, such as HAGLROS, LBX2-AS1, LINC00900, LINC01614, AC090673.1, and especially TC, while others were found to be related to TC for the first time. For instance, Li et al. demonstrated that LBX2-AS1 activated FSTL3 by binding to the TF RARα to hasten the proliferation, migration, and invasion of thyroid cancer [29]. HAGLROS was included in a prognostic model for TC, which was significantly correlated with TC recurrence [30]. Li et al. [31]constructed a prognostic model composed of seven lncRNAs, including LINC01614, AC090673.1, and LINC00900, which could serve as potential biomarkers for THCA prognosis. Nevertheless, it is necessary to validate whether this immune-related lncRNA model could be a helpful predictive indicator in TC. Many researchers are currently focused on constructing signatures with both coding genes and noncoding RNAs, which can assess the survival status of patients with malignant carcinoma [32, 33]. Unlike most traditional risk models, our newly constructed signature involved two-lncRNA pairwise comparisons and relative ranking based on gene expression entirely from the same TC patient. Although from different sequencing platforms, our prognostic model does not require gene expression data normalization. Previous studies have supported the effectiveness of this method [34, 35].

In the current study, we established an immune-related lncRNA model and evaluated its prognostic value as well as its correlation with immune cell infiltration, ICI-related genes, and TKIs in TC. First, we performed a differential coexpression analysis to identify DEirlncRNAs based on data from TCGA. LncRNA pairs were systematically identified through pairwise comparisons in the same sample without the need for data normalization. In addition, univariate analysis with Lasso regression analysis was performed on the pairs to validate the most suitable variables. Fourteen significant DEirlncRNA pairs with maximum prognostic values were determined with multiple repeats and random stimulation. Next, we used these pairs to develop the predictive risk score model. Then, we calculated not only the 3-, 5-, and 10-year AUC values of the ROC curve but also identified the optimal cutoff point of the 1-year ROC curve to separate TC patients into high- and low-risk groups. Furthermore, Kaplan‒Meier curves, time-dependent ROC curves, and Cox proportional hazards regression analysis showed that the model had independent predictive value for TC prognosis. Finally, we evaluated the relationship between this novel model and tumor-infiltrating immune cells, ICI-related molecules, and small-molecule inhibitor validity.

Immune infiltrates in the tumor microenvironment (TME) play a vital role in tumor development and progression and affect the clinical outcomes of cancer patients [36]. Dysfunction of the immune status in the TME contributes to the development and progression of cancer, and this is the basis of many immunotherapy studies. Moreover, tumor immunotherapy is now considered to have an important role in the elimination of cancer cells and sheds light on the mechanisms of cancer immune evasion, contributing to tumor outgrowth. Recent studies have suggested that lncRNAs play a central role in innate and adaptative cancer immunity regulation [37]. Immune-related lncRNA pairs as signatures are better at predicting prognosis than single lncRNAs. Therefore, it is necessary to explore more immune-related lncRNAs in tumors for future clinical practice. In this research, we carried out pairwise comparisons of a given set of immune-related lncRNAs and expression values. Thus, our prognostic signature could help address batch effects between different platforms and overcome the reprocessing and normalization of data.

Immune cell infiltration reflects the TME and reportedly impacts the outcome of TC progression. Immune-related lncRNAs are correlated with the development of TC. To explore the relationship between the prognostic model and immune-infiltrating cells, we applied seven commonly accepted methods, including TIMER [38, 39], CIBERSORT [40], XCELL [41], QUANTISEQ [42], MCP-counter, [43] EPIC [44], and CIBERSORT-ABS [45]. By integrating analyses, we found that the levels of Tregs, myeloid dendritic cells, monocytes and cancer-associated fibroblasts in the high-risk group were higher than those in the low-risk group, while the levels of neutrophils, M1 macrophages, NK cells, CD8 + , CD4 + T cells, and B cells were significantly negatively correlated with the risk of signature. These results were consistent with the findings of some previous experimental studies, which aimed to determine the correlation between each cell type and the aggressiveness of TC. For example, it has been reported that neutrophils play an antitumor role and can be beneficial to the prognosis of TC [46], which is consistent with the findings of our analysis. Our results also revealed that the abundance of Tregs was more associated with the high-risk group, which is similar to the findings of previous literature. In those studies, the levels of Tregs were higher in PTC than in multinodular goiter patients, and Tregs were consistently present in extraglandular invasion and lymph node metastasis [47, 48]. Monocytes have been observed to promote the occurrence and development of tumors, and their high density is closely related to thyroid tumor invasion and reduced survival, which was also confirmed in our study [49]. Dendritic cells (DCs) are the sentinel antigen-presenting cells (APCs) of the immune system [50], which suggests that the high level of DCs observed in the high-risk group could favor antigen presentation to T cells. Existing evidence shows that CD8 + , CD4 + T cells, and B cells play a protective role in the PTC tumor microenvironment (TME) [6]. In a wide immunohistochemical characterization of the immune network in patients with chronic lymphocytic thyroiditis concurrent with DTC, high CD8 + T lymphocyte infiltration was associated with improved disease-free survival. In addition, CD4 + T cells often differentiate into helper T cells, producing cytokines, and cooperating with CD8 + T cells to kill cancer cells [48]. A study indicated that B cells could promote carcinogenesis by inducing immunosuppression [51]. Natural killer (NK) cells are a family of innate immune cells, which play a central role in antiviral immunity and tumor immunosurveillance. A recent experimental study found that IL-12 immunotherapy could inhibit tumor growth and prolong survival by reactivating both CD8 + T and NK cells [52]. These observations can be further explored for a holistic understanding of the nuances of immune cell infiltration in the TC microenvironment.

The granzyme-perforin pathway is a primary method by which cytotoxic lymphocytes destroy cancer cells [53]. GZMA is a member of the granzyme family and is mainly secreted by cytotoxic cells, such as cytotoxic T cells and natural killer (NK) cells. PRF1 form membrane pores releasing granzymes leading to the cytolysis of the target cells [54]. We found that GZMA was highly expressed in the low-risk group, which is consistent with the higher NK cell percentage in the low-risk group (Additional file 7: Fig. S5). The ESTIMATE algorithm was used to explore the underlying mechanisms that cause PTC immune differences, and we found that the low-risk subset had higher stromal and immune scores. This finding is consistent with previous studies concluding that patients with high TME scores exhibit a stronger antitumor immune response, re likely to benefit more from immunotherapy, and survive longer [55]. Pioneering investigations have revealed that immunotherapy targeting immune checkpoints and human leukocyte antigen (HLA) provide great hope for the clinical treatment of human cancers [56]. The results showed that the expression levels of HLA-related genes were significantly higher in the low-risk group. Subsequently, we studied the interaction between the risk score and immune-related functions. The above findings further explained the reasons for the tumor-promoting status in the low-risk group and revealed the immunosuppressive microenvironment present in this group.

Recently, ICB immunotherapy has been viewed as a promising cancer therapeutic modality for malignant tumors. The identification of PD-L1 as an immunostat blockade has led to the development of several cancer immunotherapies. For RAI-refractory PTC patients, recent evidence has shown that overexpression of PD-L1 together with lymphocyte infiltration into the tumor TME is significantly associated with the effectiveness of ICB [57, 58]. Liotti et al. [59] observed a positive PD-1 effect on TC cell proliferation and migration through SHP-2 action on the Ras pathway. In this study, we found that patients in the low-risk subgroup had higher expression of common immune checkpoint molecules such as PD-1, PD-L1, LAG3, CTLA-4, PD-L2, and CD74, which are commonly expressed in human cancer. This phenomenon could indicate that low-risk patients might have a better, more beneficial response from ICB immunotherapy. Tyrosine kinase inhibitors (TKIs) are an innovative personalized strategy that target pro-oncogenic kinases, including EGFR, MET, PDGFR, VEGFR-1, VEGFR-2, RAF, FGFR, and RET. Our signature showed that the risk score was related to some of these inhibitors, such as gefitinib, sunitinib, and tipifarnib, indicating that this new model might be a novel method for assessing the efficacy of systemic therapy based on a genetic understanding of TC. In addition, some clinical trials have applied PD-1 and PD-L1 inhibitors in combination with TKIs, RAIs, or chemotherapy to manage and defeat deadly TC. Our signature may provide new insight to predict which patients are more suitable for these treatments, either alone or in combination.

To the best of our knowledge, a prognostic model based on irlncRNA pairs in TC has not been reported to date. Our predictive model is based on a 0-or-1 matrix and could be applied in an individualized manner while eliminating batch bias. In addition, our signature first combined DElncRNA pairs with ICB and TKI efficacy for analysis. Various additional methods were used to support the prognostic value and feasibility of this new model.

However, this lncRNA-based prognostic signature had several limitations. First, the establishment and validation of the model was based only on the TCGA database, which might lead to selection bias. To verify the predictive values of the risk assessment model, a larger external dataset of TC should be analyzed, preferably validated using GEO datasets. Unfortunately, no survival information of THCA could be obtained from the GEO cohort. Second, the research on the relationship between DElncRNA pairs and ICB and TKI efficacy was based on the inference of several algorithms and has not been experimentally verified. In addition, the calculation formulas of this prognostic signature may be too complex for clinical application. However, more experimental studies are still needed to validate our observations.

## Conclusion

In conclusion, we constructed an immune-related lncRNA pair model without considering the technical bias of different platforms. As such, our novel model could be used without the need to eliminate batch effects and could serve as an independent single-sample estimate of the survival risk subgroup of TC patients. The model may provide new possibilities for translation to clinical practice for TC patients and help in distinguishing those who could benefit from ICB immunotherapy and TKI therapy.

## Supplementary Information

**Additional file 1: Table S1.** A total of 1148 immune-related lncRNAs were validated by co-expression analysis (submitted as a separate EXCEL file).

**Additional file 2: Table S2.** Premier Sequences for qRT‒PCR Analysis.

**Additional file 3: Figure S1.** The expression levels of 14 single lncRNAs of DEirlncRNA pairs quantified using qRT-PCR analysis in 12 paired thyroid cancer tissues and no-tumorous samples.

**Additional file 4: Figure S2.** Boxplots indicated that (a) age, (b) survival status, (c) N stage, and (d) clinical stage were related to the risk score.

**Additional file 5: Figure S3.** Confirmation of the Signature in the Training Set.

**Additional file 6: Figure S4.** Confirmation of the Signature in the Testing Set.

**Additional file 7: Figure S5.** The Specific Correlations between Tumor Infiltrating Immune Cells and the Signature.

**Additional file 8: Table S3.** The P value of Comparing Risk Sore and Tumor Infiltrating Immune Cells.

## Data Availability

The generated and analyzed datasets of the current research are available in TCGA database (http://cancergenome.nih.gov/), and the immune-related genes were obtained from the ImmPort database (https://immport.niaid.nih.gov).

## Abbreviations

TC: Thyroid cancer
DEirlncRNA: Differentially expressed immune-related lncRNA
ICIs: Immune checkpoint inhibitors
DTC: Differentiated thyroid cancer
ICB: Immune checkpoint blockade
TKIs: Tyrosine kinase inhibitors
lncRNAs: Long noncoding ribonucleic acids
TCGA: The cancer genome Atlas
LASSO: Least absolute shrink age and selection operator
qRT-PCR: Quantitative real-time PCR
PCA: Principal component analysis
ROC: Receiver operating characteristic
TME: Tumor microenvironment
