# Supplementary material for: A novel signature to predict thyroid cancer prognosis and immune landscape using immune-related LncRNA pairs
Source: BMC Med Genomics. 2022 Aug 22;15:183. doi: 10.1186/s12920-022-01332-7 (PMC9394074; doi:10.1186/s12920-022-01332-7)
Supplement: Supplementary file 2 — Additional file 2: Table S2. Premier Sequences for qRT‒PCR Analysis. [file 12920_2022_1332_MOESM2_ESM.docx]

**Additional file 2: Table S2: Premier Sequences for qRT‒PCR Analysis**

| Premier | Sequences (5’-3’) |
| --- | --- |
| LINC00900-F  LINC00900-R  DCST1-AS1-F  DCST1-AS1-R  HAGLROS-F  HAGLROS-R  LINC01614-F  LINC01614-R  ELN-AS1-F  ELN-AS1-R  LBX2-AS1-F  LBX2-AS1-R  LINC02560-F  LINC02560-R  AC012038.2-F  AC012038.2-R  AL158206.1-F  AL158206.1-R  AC007255.1-F  AC007255.1-R  AC093585.1-F  AC093585.1-R  AC005237.1-F  AC005237.1-R  SMIM25-F  SMIM25-R  AC090673.1-F  AC090673.1-R  GAPDH-F  GAPDH-R | GGAGTTGAGGCGGAAGGATTGC  GAGGGTGTCTGGGTAGTGAGTGAG  AGGGGATGGTGATAACAGGGAGAAG  GGAGGATGGAGATGGTGGTCTGG  GAGCCACAGTGACCTTGCATTCC  AGTCTTAGCCTACTTCCTCCCACAC  ACCATTAGCAGATGCCACAGGATTC  CCAGAGACCTTGCCTTCAGGATTTG  ACCACCACCTCCCGAGTTCAAG  CCTCCATCAGCCTCAAATCTCCAAG  GCCTTCTCACCACCGCTGAATTAG  GCCTTATTCTTCCTGCCTCACACTC  CGCATTCTCCGTCTTAACCAGCTC  GGCACAACTCCAGCTCTGAAGAAG  GGAAGTGCTGCCCAAAGACA  GCCGCAGGAATTAACCAATATGA  AGGTCATGGTTGTGGTTACTTGGC  GCACTCTTCACTGGACAGGCATAG  GTCTCCTCCCATCCTCACCACTG  AGCTCACCCACACCTGTCTTCC  TGGCTCAGGAGGAAGCACAGAG  GTCAAAGCAGGGAACCGCAGAG  ACCTTGGTGGCCTCATCCTCTG  AATCGTCTCATAGCGGTGGTGTTG  GACGCAGGCTCTAACAAGTGGATAC  GGGAGACAATCAAGCAGGGAACAG  GGAAATCCAAGGAAGATCGGGTGAG  AGGTCCAAGCAATGCCAGAACAG  GTCTCCTCTGACTTCAACAGCG  ACCACCCTGTTGCTGTAGCCAA |
