# Supplementary material for: A novel signature to predict thyroid cancer prognosis and immune landscape using immune-related LncRNA pairs
Source: BMC Med Genomics. 2022 Aug 22;15:183. doi: 10.1186/s12920-022-01332-7 (PMC9394074; doi:10.1186/s12920-022-01332-7)
Supplement: Supplementary file 3 — Additional file 3: Figure S1. The expression levels of 14 single lncRNAs of DEirlncRNA pairs quantified using qRT-PCR analysis in 12 paired thyroid cancer tissues and no-tumorous samples. [file 12920_2022_1332_MOESM3_ESM.docx]

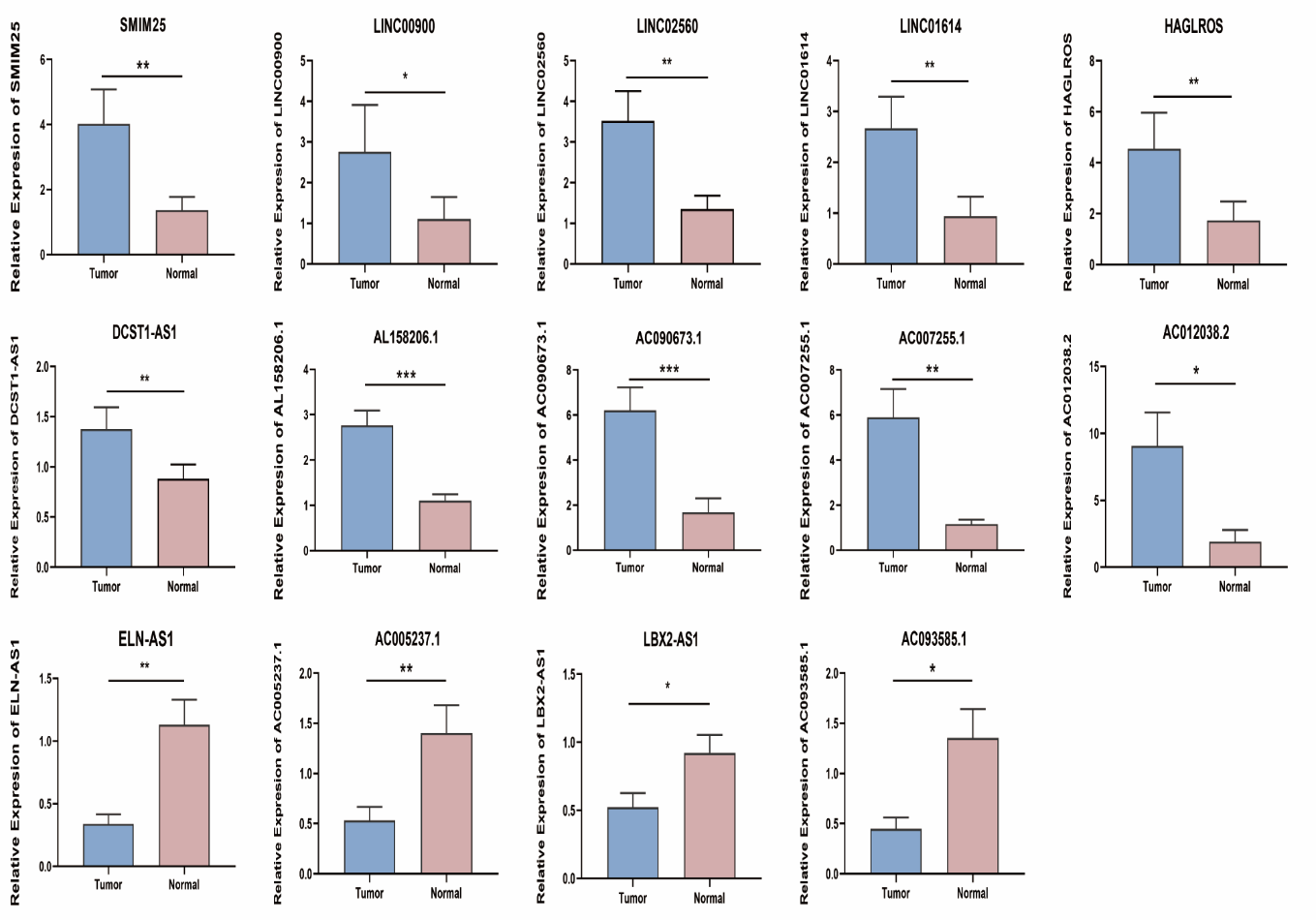


**Additional file 3: Figure S1**: The expression levels of 14 single lncRNAs of DEirlncRNA pairs quantified using qRT-PCR analysis in 12 paired thyroid cancer tissues and no-tumorous samples
