# Supplementary material for: A novel signature to predict thyroid cancer prognosis and immune landscape using immune-related LncRNA pairs
Source: BMC Med Genomics. 2022 Aug 22;15:183. doi: 10.1186/s12920-022-01332-7 (PMC9394074; doi:10.1186/s12920-022-01332-7)
Supplement: Supplementary file 4 — Additional file 4: Figure S2. Boxplots indicated that (a) age, (b) survival status, (c) N stage, and (d) clinical stage were related to the risk score. [file 12920_2022_1332_MOESM4_ESM.docx]

**
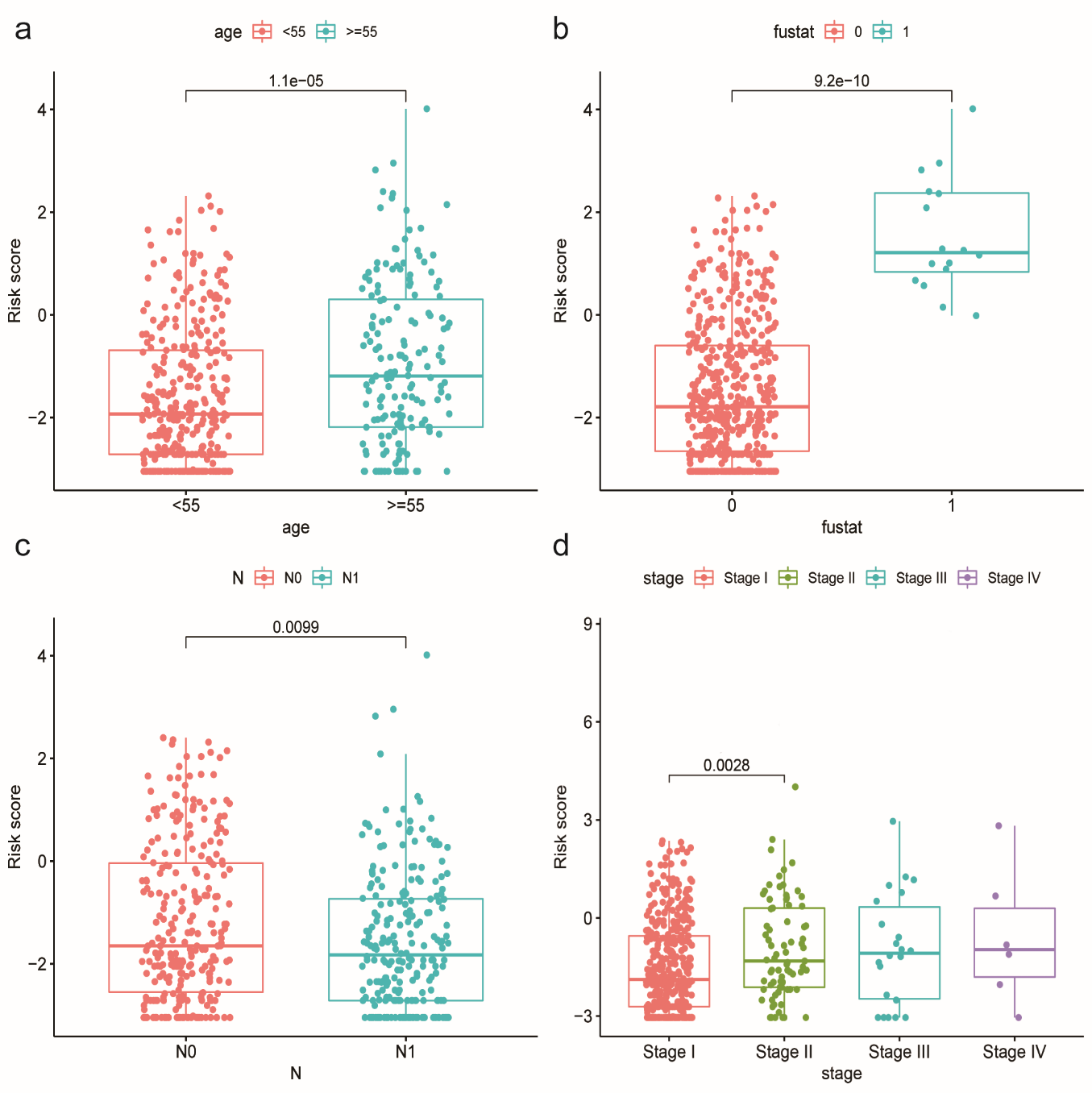
**

**Additional file 4:Figure S2:** Boxplots indicated that (a) age, (b) survival status, (c) N stage, and (d) clinical stage were related to the risk score.
