# Supplementary material for: A novel signature to predict thyroid cancer prognosis and immune landscape using immune-related LncRNA pairs
Source: BMC Med Genomics. 2022 Aug 22;15:183. doi: 10.1186/s12920-022-01332-7 (PMC9394074; doi:10.1186/s12920-022-01332-7)
Supplement: Supplementary file 5 — Additional file 5: Figure S3. Confirmation of the Signature in the Training Set. [file 12920_2022_1332_MOESM5_ESM.docx]

**
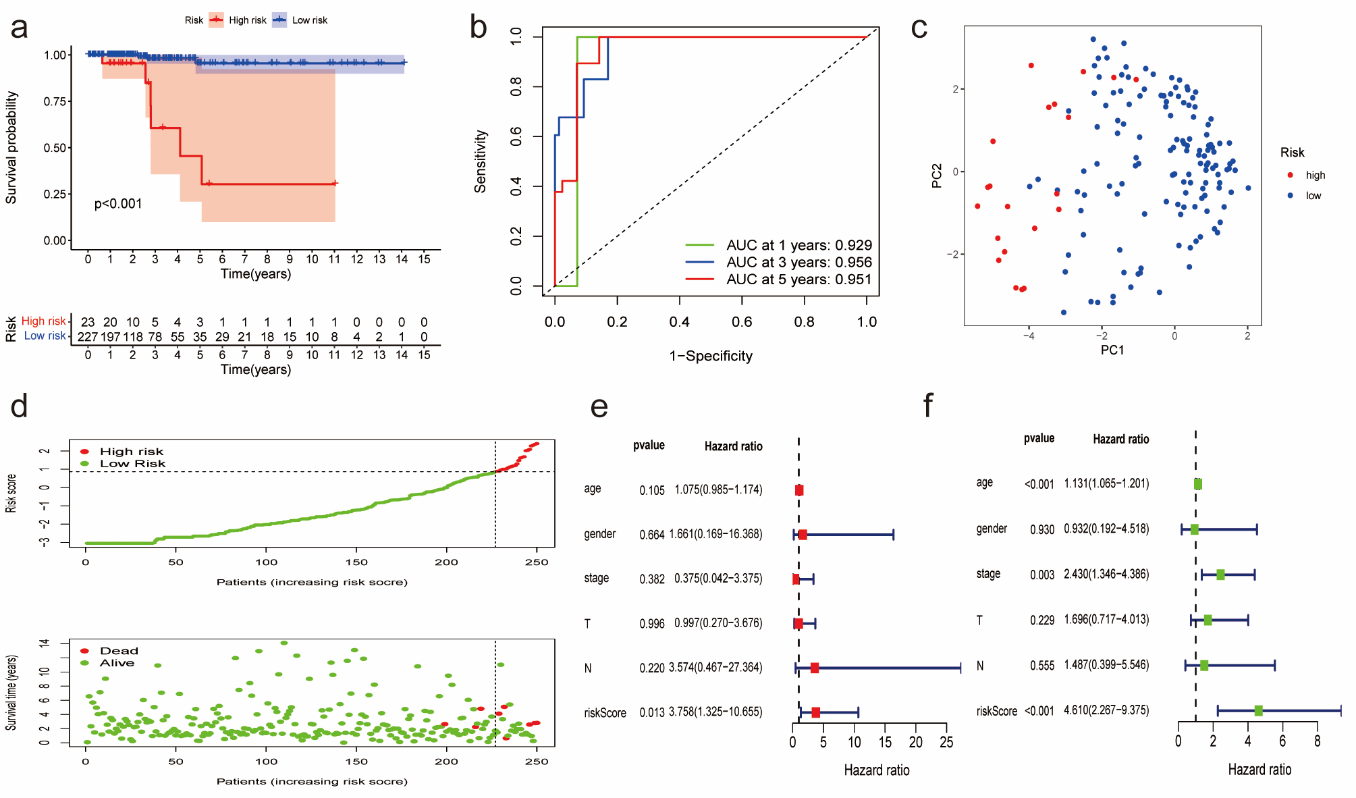
**

**Additional file 5:Figure S3: Confirmation of the Signature in the Training Set.**

(a):Kaplan‒Meier curve presenting survival in the high-risk and low-risk sets. (b): ROC analysis of the risk scores for overall prognosis prediction. The 1- (red), 3- (green), and 5-year (blue) ROC curves of the model suggested that all AUC values were over 0.90. (c): PCA plot of the lncRNA model. (d): Distribution of lncRNA model risk score and survival status of TC patients in the subgroups. (e)(f): Univariate and multivariate Cox regression analyses were applied to identify prognostic factors associated with TC.
