# Supplementary material for: A novel signature to predict thyroid cancer prognosis and immune landscape using immune-related LncRNA pairs
Source: BMC Med Genomics. 2022 Aug 22;15:183. doi: 10.1186/s12920-022-01332-7 (PMC9394074; doi:10.1186/s12920-022-01332-7)
Supplement: Supplementary file 7 — Additional file 7: Figure S5. The Specific Correlations between Tumor Infiltrating Immune Cells and the Signature. [file 12920_2022_1332_MOESM7_ESM.docx]

**
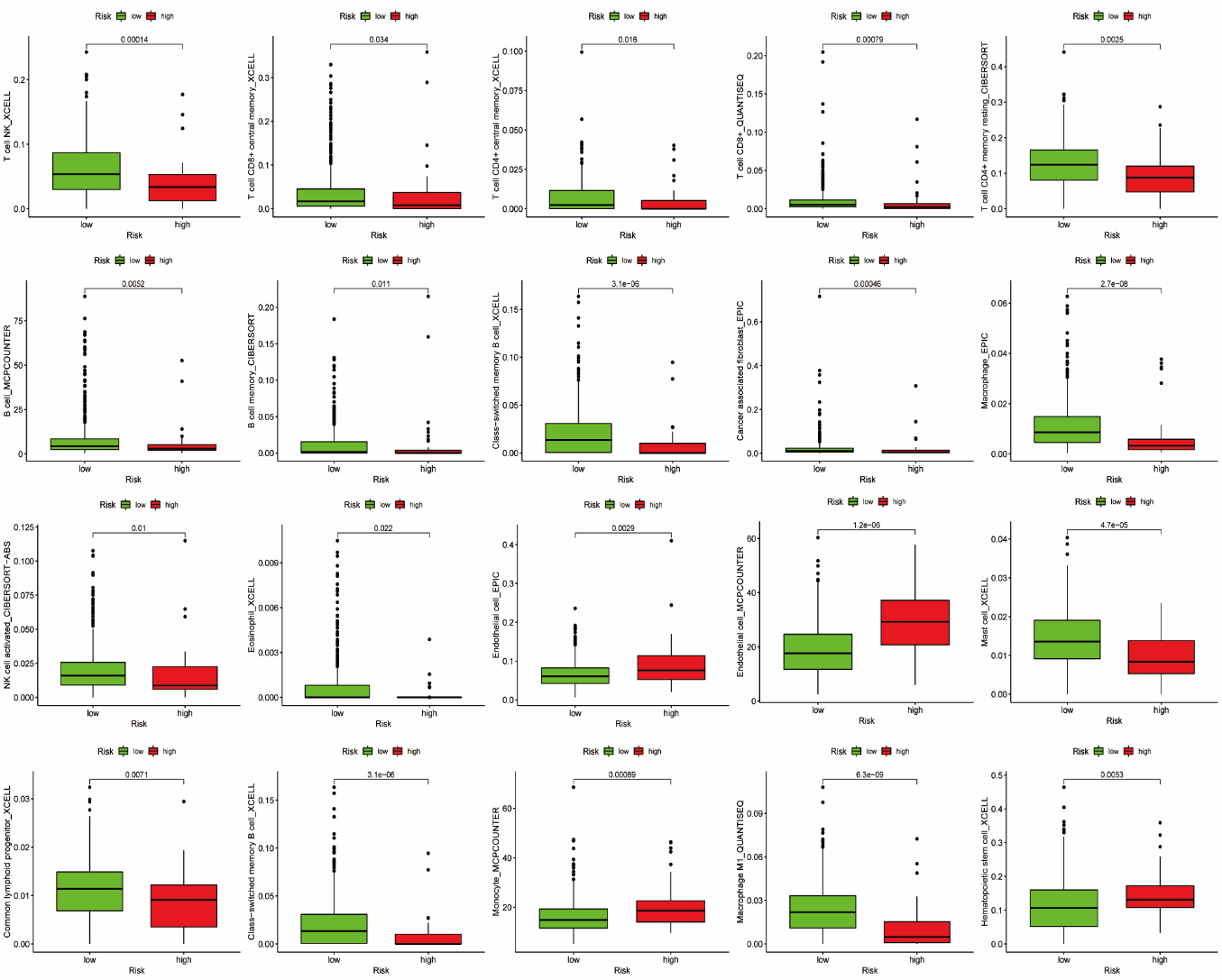
**

## Additional file 7: Figure S5: The Specific Correlations between Tumor Infiltrating Immune Cells and the Signature.
