# Supplementary material for: A novel signature to predict thyroid cancer prognosis and immune landscape using immune-related LncRNA pairs
Source: BMC Med Genomics. 2022 Aug 22;15:183. doi: 10.1186/s12920-022-01332-7 (PMC9394074; doi:10.1186/s12920-022-01332-7)
Supplement: Supplementary file 8 — Additional file 8: Table S3. The P value of Comparing Risk Sore and Tumor Infiltrating Immune Cells. [file 12920_2022_1332_MOESM8_ESM.docx]

## Additional file 8: Table S3: The *P* value of Comparing Risk Sore and Tumor Infiltrating Immune Cells.

| Immune cell | *P* value | Immune Cell | *P* value |
| --- | --- | --- | --- |
| T cell CD4+_TIMER  T cell CD4+ Th1_XCELL  T cell CD4+ Th2_XCELL  B cell naïve _CIBERSORT  B cell naïve _CIBERSORT-ABS  Monocyte _CIBERSORT  Monocyte _CIBERSORT-ABS  Myeloid dendritic cell resting_ CIBERSORT  Myeloid dendritic cell resting_ CIBERSORT-ABS  Myeloid dendritic cell_ MCPCOUNTER  T cell CD8+ naïve _XCELL  T cell CD8+_XCELL  Neutrophil _CIBERSORT  Neutrophil _CIBERSORT-ABS  Mast cell activated_ CIBERSORT-ABS  Mast cell resting_ CIBERSORT-ABS  Mast cell _XCELL  B cell_ QUANTISEQ  B cell plasma _XCELL  Macrophage M2_QUANTISEQ  T cell CD4+ (non-regulatory) _QUANTISEQ  Myeloid dendritic cell_ QUANTISEQ  uncharacterized cell_ QUANTISEQ  cytotoxicity score_ MCPCOUNTER  uncharacterized cell _EPIC | 0.0040  0.0000  0.0000  0.1129  0.0304  0.0124  0.0032  0.0009  0.0095  0.0000  0.0340  0.0059  0.0140  0.0106  0.0140  0.0041  0.0219  0.0341  0.0000  0.0003  0.0000  0.0000  0.0000  0.0000  0.0005 | Monocyte_ MCPCOUNTER  Macrophage/Monocyte_ MCPCOUNTER  Endothelial cell_ MCPCOUNTER  T cell CD4+ memory_ XCELL  T cell CD4+ naive_ XCELL  T cell CD4+ naïve_ CIBERSORT  T cell CD4+ naïve _CIBERSORT-ABS Neutrophil _TIMER  Neutrophil QUANTISEQ  Mast cell activated CIBERSORT  Mast cell resting CIBERSORT  Eosinophil_ XCELL  Granulocyte-monocyte progenitor_ XCELL  Hematopoietic stem cell_ XCELL  T cell NK_XCELL  Plasmacytoid dendritic cell XCELL  T cell regulatory QUANTISEQ  T cell regulatory (Tregs)_XCELL  B cell EPIC  T cell CD4+_EPIC  T cell CD8+_EPIC  Macrophage _EPIC  NK cell _EPIC  T cell CD4+ (non-regulatory) _XCELL  T cell CD4+ central memory _XCELL | 0.0000  0.0000  0.0155  0.0241  0.0064  0.0349  0.0351  0.0404  0.0344  0.0077  0.0063  0.0000  0.0042  0.0451  0.0060  0.0000  0.0141  0.0001  0.0020  0.0324  0.0004  0.0000  0.0000  0.0157  0.0000 |
